# Microplastic-induced alterations in water flow and solute transport dynamics in soil

**DOI:** 10.1038/s41598-025-30476-6

**Published:** 2025-12-01

**Authors:** Milad Aminzadeh, Tanmay Kokate, Ali Usman Chaudhry, Harris Rabbani, Branko Bijeljic, Martin J. Blunt, Nima Shokri

**Affiliations:** 1 Institute of Geo-Hydroinformatics, Hamburg University of Technology, Hamburg, Germany; 2 United Nations University Hub on Engineering to Face Climate Change at the Hamburg University of Technology, United Nations University Institute for Water, Environment and Health (UNU-INWEH), Hamburg, Germany; 3 College of Science and Engineering, Hamad Bin Khalifa University (HBKU), Doha, Qatar; 4 Department of Earth Science and Engineering, Imperial College, London, UK

**Keywords:** Microplastics, Sandy soil, Microfluidic experiment, Porous media, Hydraulic conductivity, Solute transport, Environmental sciences, Hydrology

## Abstract

The growing use of plastic-based practices in agriculture has led to a significant accumulation of plastic waste in soil. Microplastics (MPs) increasingly threaten soil health and fertility by disrupting its physical and chemical environment, and impairing essential ecological functions. We conducted laboratory column measurements combined with microfluidic experiments to assess the effects of MPs on water flow and solute transport in soil, key processes for sustaining soil water and nutrient availability and thus crop growth and yield. Changes in hydraulic conductivity and solute breakthrough curves in sandy soils were investigated in the presence of varying concentrations of polyethylene (PE) and polyvinylchloride (PVC) microplastics. Alterations in pore structure and clogging of pore throats by MPs, as further evidenced through confocal and fluorescence microscopy of synthesized porous media, led to 39% and 74% reductions in hydraulic conductivity of sand samples containing 5% PVC and 5% PE, respectively. Solute transport experiments using a brine tracer revealed broader breakthrough curves in the presence of MPs. Overall, the enhancement of pore-scale flow heterogeneity driven by the development of preferential flow paths and the formation of low-permeability zones increased hydrodynamic dispersion and resulted in both early breakthrough and delayed transport of the tracer within the soil column.

## Introduction

The rising demands of a rapidly growing population have led to a significant increase in plastic production over the past few decades^1,2^. The agricultural sector has adopted various plastic-based practices such as plastic mulching and seed coating to enhance crop yields and improve soil and water management to meet the need for higher food production^3,4^. Although these methods provide short-term agronomic benefits, their widespread use has unintentionally contributed to increased plastic contamination in the soil environment^5,6^. Soil contamination by so-called agroplastics is expected to intensify as global demand for greenhouse, mulching, and silage plastic films is projected to reach 9.5 million tons by 2030, a more than 50% increase compared to 2018^7,8^.

Recent studies have revealed that plastic particles adversely affect soil health and impair its vital ecological functions^9,10^. Investigating more than 5,100 global observations, Chen et al.^11^ showed that presence of plastics in soil significantly hinders seed germination and crop growth, reduces microbial diversity, and intensifies greenhouse gas emissions by altering carbon and nitrogen cycling processes. Microplastic particles, smaller than 5 mm in size^12^, can significantly alter the physical and chemical environment of soil. Their presence in pore spaces disrupts soil aggregation and pore network connectivity^13–16^. These structural changes affect soil aggregates and shift pore-size distribution, thereby modifying infiltration and water retention characteristics as shown by Liu et al.^17^ and Wang et al.^18^. The intrinsic properties of microplastics (e.g., polymer composition, density, and hydrophobicity) together with surface transformations during weathering (e.g., changes in surface charge and roughness) influence both soil physical and chemical environment. These modifications affect soil wettability and water repellency^19,20^, as well as key chemical properties such as pH, cation exchange, and nutrient binding^21–23^. Neutron imaging of sand columns containing microplastics by Cramer et al.^24^ demonstrated that local reductions in wettability caused by MPs impede capillary rise and restrict water distribution, thereby reshaping soil flow pathways. Furthermore, changes in surface charge and sorption capacity affect ion exchange and nutrient mobility, directly modifying solute breakthrough and dispersion patterns^25,26^. Microplastic surfaces also interact strongly with dissolved organic matter and agrochemicals thus promoting contaminant retention or co-transport^27^. Such combined physical and chemical modifications influence flow characteristics, sorption, and dispersion processes, ultimately altering the movement and availability of water, nutrients, and agrochemicals within the soil profile^26,28^.

Despite increasing attention to soil microplastic pollution, most studies have focused on identifying the occurrence and distribution of MPs^29,30^, evaluating their migration and retention in soil^31–33^, or assessing biological and chemical responses in microplastic-contaminated soils^22,34^. Consequently, fewer investigations have examined how microplastics modify fundamental hydrodynamic processes, particularly the coupled behavior of water flow and solute transport in soil. While previous transport studies have primarily addressed the mobility of specific chemicals or ions in the presence of microplastics^35,36^, the extent to which MPs influence soil flow regimes and solute breakthrough characteristics remains less understood. In particular, the combined effects of microplastic properties (e.g., size, shape, and polymer type) and alterations in pore connectivity on solute dispersion, breakthrough behavior, and leaching pathways are largely unexplored. This knowledge gap is especially critical considering the fundamental role of transport processes in maintaining soil health, nutrient cycling, agrochemical leaching, and overall ecosystem resilience with rising microplastic contamination^37–40^. This study thus aims to investigate the impact of microplastics on water flow characteristics and solute transport through the soil profile. By integrating soil column experiments with microfluidic visualization, we link pore-scale processes with macroscale soil responses to provide mechanistic insights into how MPs disrupt pore network and consequently alter flow and solute transport as the key processes which influence soil functionality and agricultural sustainability.

## Materials and methods

### Materials

**Fig. 1:**
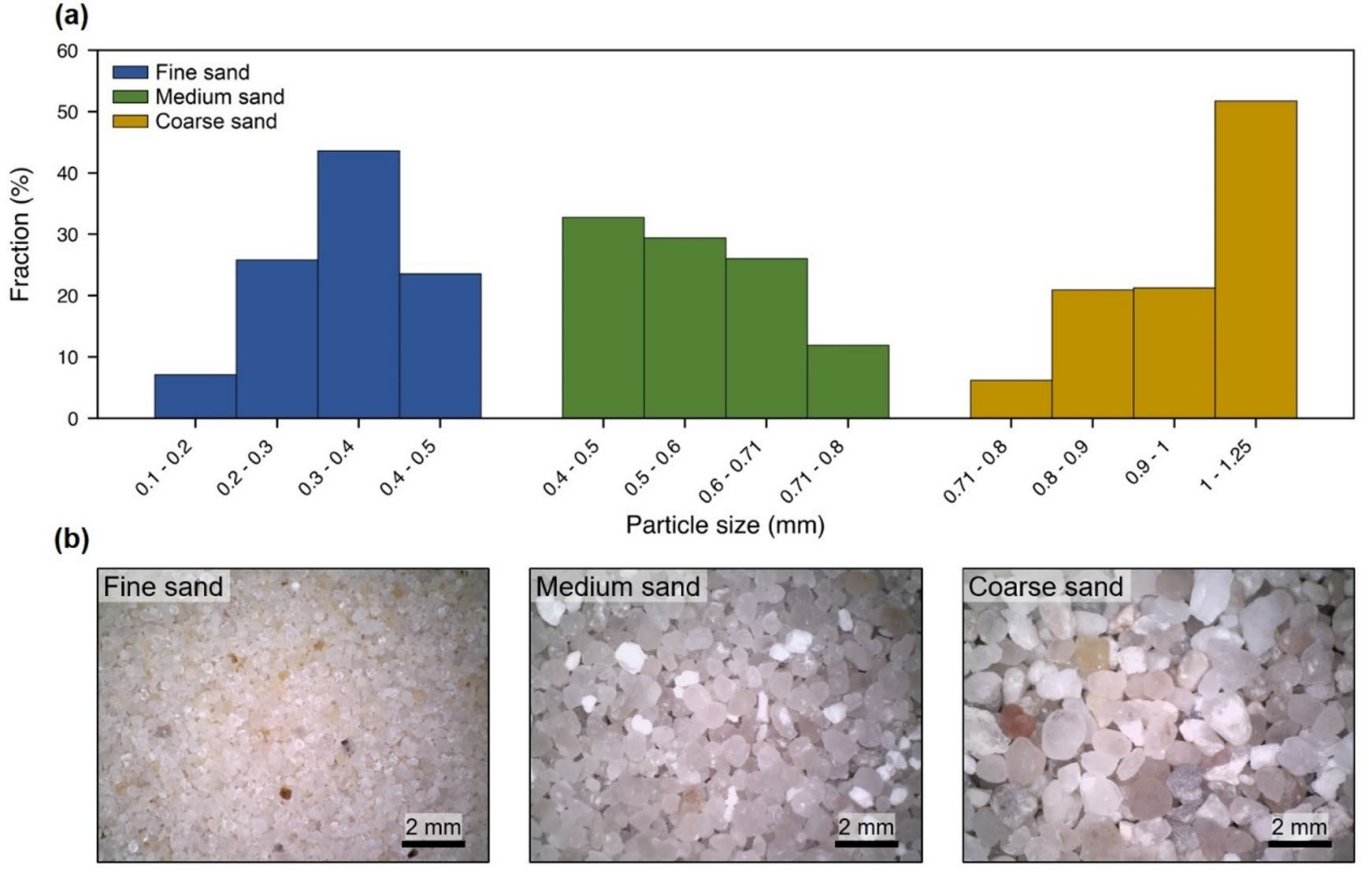
(**a**) Particle size distribution of fine, medium, and coarse sandy soils obtained from sieve analysis. (**b**) Surface images of the sand samples.

The experiments were conducted using sandy soils with fine, medium, and coarse sand fractions, corresponding to particle size ranges of 0.1–0.5 mm, 0.4–0.8 mm, and 0.7–1.2 mm, respectively, and density of ~ 2.4–2.6 g/cm^3^. Figure 1 depicts particle size distribution of sandy soils determined based on the sieving method. Two types of microplastic were used in our experiments: ultra-high molecular weight polyethylene (PE) with particles between 34 and 50 *µ*m and density of 0.94 g/cm^3^, and low molecular weight polyvinylchloride (PVC) with particle sizes ranging from 80 to 200 *µ*m and density of 1.4 g/cm^3^ (Sigma-Aldrich). PE and PVC are among the most commonly detected polymers across diverse soil types^41^.

To evaluate the influence of microplastics on transport processes in soil, hydraulic conductivity and solute transport experiments were conducted at MP concentrations of 2% and 5% (by mass), thoroughly mixed with dry sand. Similar concentration ranges have also been adopted in prior studies investigating the impact of microplastics on various inherent soil properties and transport processes^18,42–45^. Such high concentrations of MPs have been reported by Fuller & Gautam^46^ detecting concentrations as high as 6.7% (mass basis) in soil samples from an industrial area. Dry mass and volume of samples were utilized to determine bulk density, while their porosity was obtained using the volumetric saturation method outlined by Missimer & Lopez^47^. All measurements were repeated three times to ensure reproducibility.

### Microfluidic experiments to evaluate microplastic distribution in pore spaces

To investigate the pore-scale clogging behavior of microplastic particles within porous media, we conducted a series of microfluidic experiments using synthesized microchips. During the microfluidic experiments the images were obtained using fluorescence (Nikon Eclipse Ti2) and confocal microscopes (Leica Stellaris 5). These imaging techniques allowed us to examine the distribution and accumulation of microplastics, particularly in relation to pore structures and channels of varying dimensions.

**Fig. 2:**
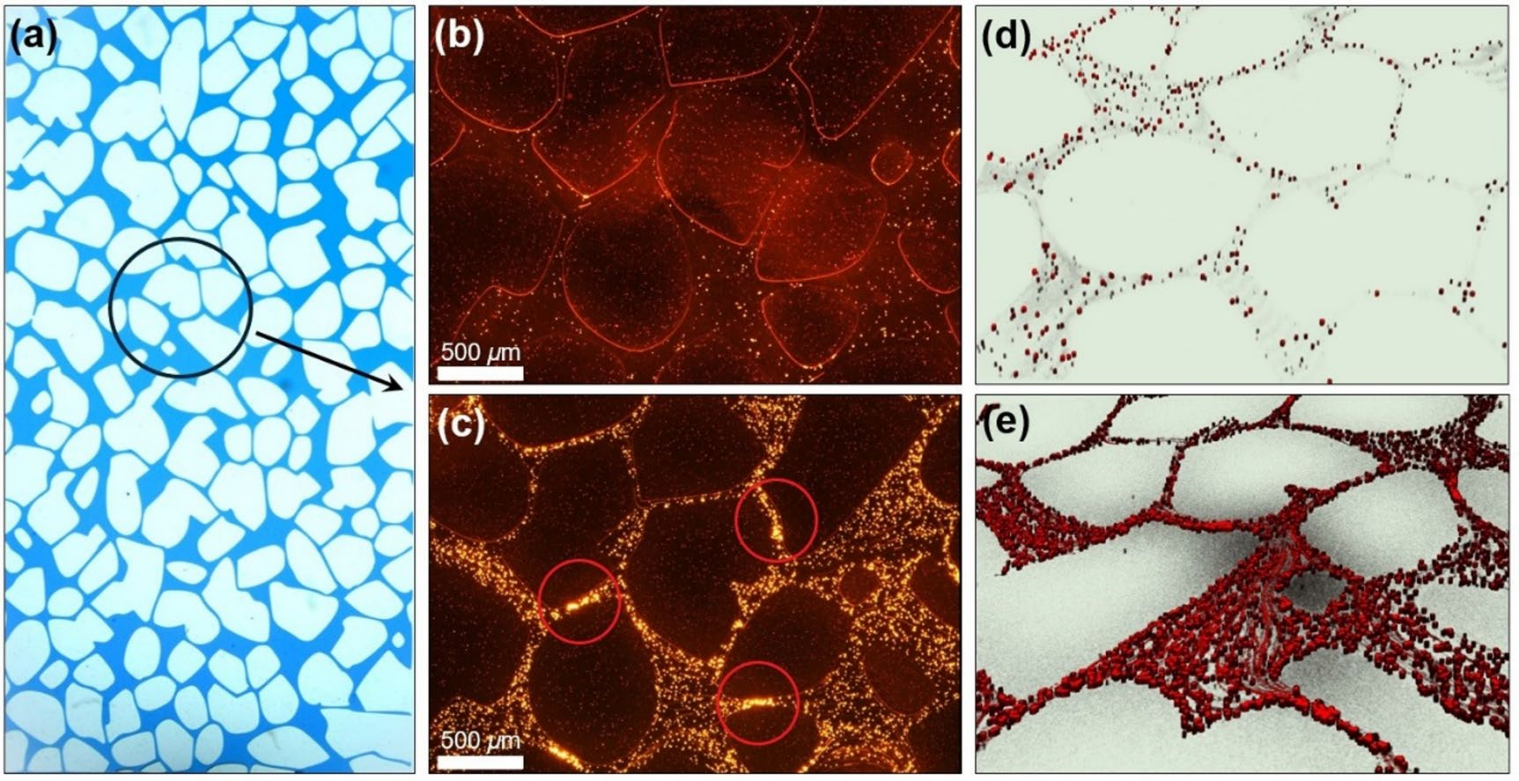
(**a**) Design of the porous medium to mimic and visualize the distribution dynamics of MPs in pore spaces. Particles are depicted in white, while pore spaces are represented in blue. Deposition pattern of fluorescence microplastics in porous media under a flow rate of 0.35 ± 0.10 *µ*L/min, depicted in fluorescence microscopic images (**b**) and (**c**) at concentrations of 50 mg/L and 100 mg/L, respectively, and in confocal microscopic images (**d**) and (**e**) at concentrations of 50 mg/L and 100 mg/L, respectively (images taken at 4X). The circles in (**c**) mark examples of microplastic deposition at pore throats.

Fluorescent microplastic particles (excitation wavelength of 575 nm and an emission peak at 607 nm), 1–5 µm, purchased from Cospheric, USA, were sonicated for 30 min to ensure homogeneous dispersion and minimize pre-aggregation. The suspension was immediately loaded into a reservoir tank connected to the pressure controller to achieve the target flow rate. The micoroplastic experiments were conducted on a layout which was created using computer-aided design (Fig. 2a) incorporating the desired porous media features^48^. The polydimethylsiloxane (PDMS) porous media microchip was synthesized using soft lithography as mentioned previously in Fujii^49^. Prior to injection into the porous media, MP particles were dispersed in water by sonication (for 30 min) to ensure a uniform suspension. The experiments were performed at a flow rate of 0.35 ± 0.10 *µ*L/min using a reservoir tank connected to an ElveFlow pressure system (Model OB1 Mk3 200). Imaging was performed 40 min after the flow injection using both fluorescence and confocal microscopy, under two microplastic concentrations of 50 mg/L and 100 mg/L.

### Saturated hydraulic conductivity

The saturated hydraulic conductivity (*K*_*s*_) of pure sand and samples including microplastics were measured using the constant head technique, following the procedure described by Hillel^50^. Sand samples were packed into a Plexiglass column with 20 cm height and 8 cm diameter. Once steady-state conditions were reached, (tap) water flow through the column was recorded and *K*_*s*_ values were determined based on the Darcy’s law:

1 $$\:{K}_{s}={q}_{w}\:\left(\frac{L}{\varDelta\:H}\right)\:$$

where *q*_*w*_ is the water flux (m/s), *L* is the length of the sand column (m), and *ΔH* is the hydraulic head (m). Each measurement of saturated hydraulic conductivity was repeated three times to ensure replicability of the results.

### Solute transport in soil

Solute transport experiment was conducted using a step input condition to investigate the transport of dissolved substances through a sand-filled Plexiglass column (height: 20 cm, diameter: 8 cm), with and without microplastics. The NaCl solution with concentration of 2 g/L and electrical conductivity (EC) of 4,000 *µ*S/cm was introduced at the column inlet using a Mariotte bottle (to maintain a constant head), replacing the initial background (tap) water that had been flowing from another Mariotte bottle (with an identical head) into the soil column. Effluent samples were collected at regular intervals to monitor NaCl concentration over time using an EC probe (Senseca, G1409) and calculate the breakthrough curve (BTC). The zeta potential of microplastics and quartz sand samples under different salinity conditions were determined using a zeta potential analyzer (SurPASS3, Anton Paar).

Solute transport in isotropic soil under one-dimensional steady-state flow, assuming a linear adsorption isotherm is described by the Advection-Dispersion Equation (ADE)^51^:

2 $$\:R\frac{\partial\:c}{\partial\:t}=D\frac{{\partial\:}^{2}c}{\partial\:{x}^{2}}-v\frac{\partial\:c}{\partial\:x}\:\:$$

in this equation, *c* represents the solute concentration (g/L) as a function of time *t* (s) and depth *x* (m); *R* is the retardation factor (-); *D* is the hydrodynamic dispersion coefficient (m^2^/s) accounting for both dispersion and diffusion; and *v* is the average pore water velocity (m/s). The analytical solution of Eq. (2) yields the effluent concentration at the outlet of the soil column with length *L* as^52^:

3 $$\:\frac{c\left(L,t\right)-{c}_{i}}{{c}_{0}\:-{c}_{i}}=\frac{1}{2}\text{erfc}\left(\frac{RL-vt}{\sqrt{4RDt}}\right)+\frac{1}{2}\text{exp}\left(\frac{vL}{D}\right)\text{erfc}\left(\frac{RL+vt}{\sqrt{4RDt}}\right)$$

here *c*_*0*_ denotes the influent solute concentration (with an EC of 4,000 *µ*S/cm), while *c*_*i*_ represents the initial concentration within the soil column (measured at an EC of 230 *µ*S/cm). Transport parameters (*R* and *D*) were estimated by fitting the ADE to the measured breakthrough curves. Parameter optimization was performed in Excel using the Solver add-in (GRG Nonlinear) by minimizing the sum of squared residuals between measured and calculated concentrations.

## Results and discussion

### Microscopic imaging of microplastic distribution

Observations through fluorescence microscopy (Fig. 2b and c) reveal a random distribution of microplastic particles within the pore space, with certain particles adhering to pore bodies. Additionally, aggregation of microplastic particles is evident across both concentrations (50 and 100 mg/L), with higher concentrations exhibiting pronounced aggregation, resulting in the clogging of pore channels (marked in Fig. 2c). The significant abundance of microplastics at higher concentrations within the larger pore space implies that the pressure loss during sudden expansion is aided by the reduced velocity, thereby extending the duration of particle deposition. Doubling the concentration of MPs resulted in an enhanced accumulation of microplastics throughout the porous medium with more clogged channels, highlighting the influence of concentration on MP behavior within the medium. Moreover, due to the relatively low flow rate of water (i.e., 0.35 *µ*L/min), the microplastics experienced reduced shear stress during the migration process. This prolonged the deposition time of MPs and facilitated their easier accumulation. Furthermore, the hydrophobicity among MP particles can lead to the compression and thinning of the double electric layer under specific conditions, resulting in the shielding of surface charge. This reduction in electrostatic repulsion between particles facilitates the coagulation process of microplastic particles^53^. Our findings of concentration-dependent aggregation and pore clogging agree with pore-scale studies showing that microplastics accumulate preferentially at pore throats and channel expansions^54^. Similar patterns were observed in column experiments simulating aquifer recharge, where retention and clogging altered with particle load and flow conditions^53^.

The 3D confocal microscopic images depicted in Fig. 2d and e exhibit various arrangements of microplastics corresponding to different concentrations. The results suggest that the distribution and accumulation of MPs within the porous media are influenced by their concentration levels, with distinct patterns emerging at varying concentrations. At lower concentrations of microplastics, the particles appear to be distributed along the grain bodies. Furthermore, they seem to constrict the channels, accumulating on both sides of them, while a notable concentration of microplastics is visible at the pore throats (restrictions). Additionally, an observation regarding the height of the microplastic particles reveals that in proximity to the pore throat, the particles appear to be in the ongoing process of deposition. In contrast to the low concentration (50 mg/L), observations indicate that as the concentration of microplastics increases, their distribution within the porous medium becomes more concentrated, potentially leading to greater accumulation as can be seen in the Fig. 2e. The random distribution of MPs for both concentrations showed that microplastics did not migrate through preferential channels and deposited throughout the porous medium.

### Effect of MPs on soil hydraulic conductivity

**Fig. 3:**
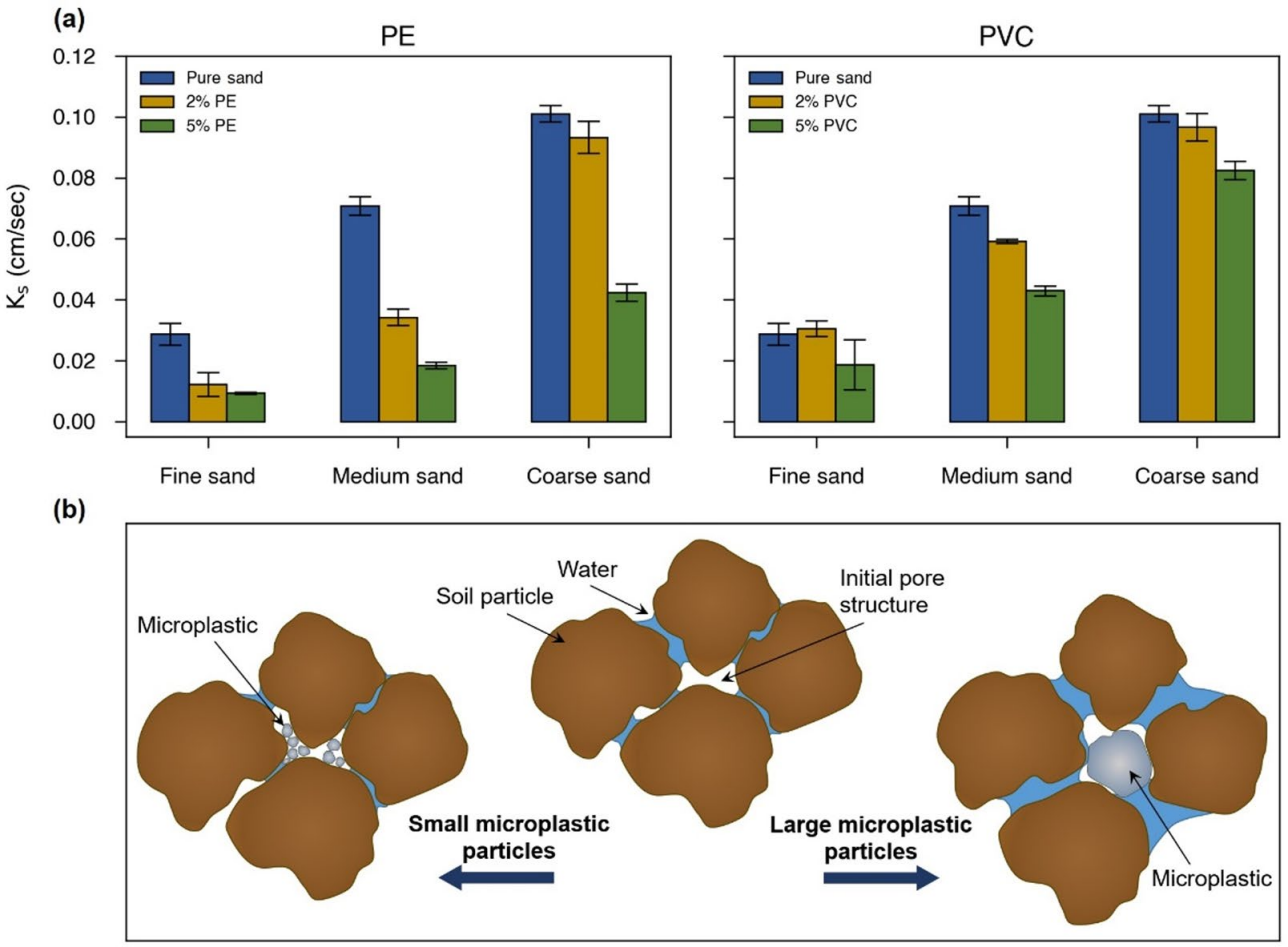
(**a**) Variation of saturated hydraulic conductivity with changes in PE and PVC concentrations for fine, medium, and coarse sandy soils measured using tap water (EC = 230 *µ*S/cm). (**b**) Schematic illustration showing how MP particle size influences pore structure: smaller MP particles relative to sand grains tend to clog pore throats, whereas larger MP particles can alter the pore space by wedging apart the grains.

Figure 3a depicts the effects of microplastics on the saturated hydraulic conductivity of fine, medium, and coarse sandy soils (measured using tap water at EC = 230 *µ*S/cm). As expected, saturated hydraulic conductivity generally increases with increase in pore spaces associated with larger particles from fine to coarse samples. Overall, hydraulic conductivity decreased with increasing concentrations of microplastics. The effect was particularly evident in medium sand, where hydraulic conductivity was reduced by 39% and 74% in samples containing 5% PVC and 5% PE, respectively, indicating a stronger impact of PE particles on soil permeability. Such reductions can be primarily attributed to the accumulation of MP particles within the soil matrix as evidenced by Fig. 2, effectively filling the pores and diminishing the overall porosity and flow connectivity in the samples (Table 1).

**Table 1:** Porosity and bulk density of sand samples at 0%, 2% and 5% concentration of PE and PVC microplastics.

	Fine sand		Medium sand		Coarse sand	
	Porosity	Bulk density (g/cm^3^)	Porosity	Bulk density (g/cm^3^)	Porosity	Bulk density (g/cm^3^)
Pure sand	0.37	1.54	0.38	1.48	0.39	1.50
2% PE	0.34	1.48	0.36	1.45	0.36	1.49
5% PE	0.32	1.37	0.33	1.46	0.34	1.50
2% PVC	0.36	1.48	0.37	1.47	0.37	1.51
5% PVC	0.35	1.43	0.36	1.48	0.34	1.44

Electrostatic interactions between soil grains and microplastic particles likely contribute to the observed changes in saturated hydraulic conductivity. As shown in Table 2, both sand grains and MPs exhibit negative zeta potentials (at EC of 230 *µ*S/cm), causing repulsive forces that influence particle mobility within the pore network. While PE with higher negative zeta potential (− 35.43 mV) remains more dispersed and can block smaller pores more effectively (consistent with the larger hydraulic conductivity reductions observed), PVC having weaker zeta potential (− 9.42 mV) tends to attach to sand grains thus promoting local pore obstruction. These electrostatic effects, together with particle size help to explain the consistent decline in hydraulic conductivity with increasing MP concentration.

**Table 2:** Zeta potential of quartz sand samples and microplastics measured under two different EC conditions, i.e., 230 *µ*S/cm (tap water) and 4,000 *µ*S/cm (NaCl solution) at 25 ± 1 °C.

	EC (µS/cm)	pH	Zeta potential (mV)
Fine sand	230	8.2	-26.45 ± 1.90
	4000	8.9	-57.37 ± 5.99
Medium sand	230	8.3	-22.35 ± 1.61
	4000	6.7	-24.35 ± 0.11
Coarse sand	230	8.4	-15.84 ± 2.62
	4000	6.5	-36.57 ± 1.03
PE	230	8.6	-35.43 ± 0.12
	4000	8.5	-55.69 ± 0.37
PVC	230	8.4	-9.42 ± 3.05
	4000	7.1	-5.45 ± 0.90

In fine sandy soil, the addition of 2% PVC microplastics resulted in a slight increase (6.5%) in saturated hydraulic conductivity. This enhancement could be attributed to the size range of the PVC particles (80–200 *µ*m) which overlaps with the finer fraction of the sand particle size distribution (100–500 *µ*m). As illustrated schematically in Fig. 3b, the PVC particles alter the pore geometry by partially replacing sand grains and changing pore spaces resembling flow in a deformable porous medium. These changes potentially improve the continuity of the pore network within the soil matrix^15^. However, a further increase in PVC concentration to 5% led to excessive particle accumulation which blocked pore channels and impeded flow, resulting in reduction of saturated hydraulic conductivity relative to the pure sand sample.

### Influence of MPs on transport of solutes in soil

**Fig. 4:**
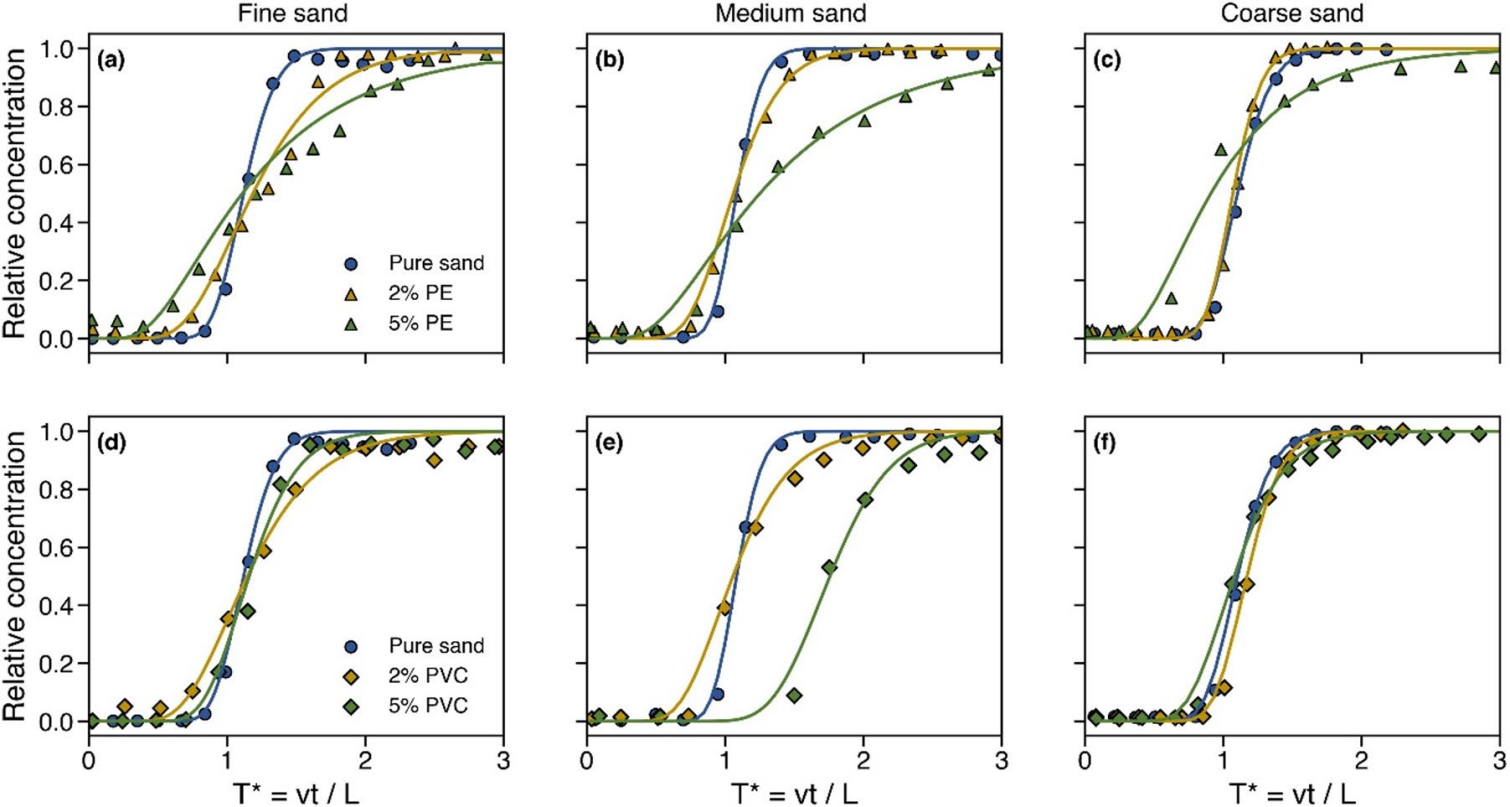
Changes in the measured (symbols) and modeled (lines) breakthrough curves of brine transport in fine, medium, and coarse sandy soils with various concentrations of PE (**a-c**) and PVC (**d-f**). T^*^ is the pore volume, v is the pore velocity, t is the elapsed time, and L is the length of the soil column.

The measured and fitted breakthrough curves for the sand samples with various concentrations of PE and PVC are depicted in Fig. 4. We observed earlier solute arrival for the 2% and 5% PE samples compared to pure fine sand (Fig. 4a). However, a delay was noted in reaching the concentration peak, after approximately 2 pore volumes (PV) for 2% PE and 3 PV for 5% PE. Figure 4b and c similarly show earlier solute arrival and a pronounced delay in peak concentration for the 5% PE sample, whereas the 2% PE sample exhibited behavior comparable to both pure medium and pure coarse sand samples. At 2% concentration in Fig. 4d, PVC particles (80–200 *µ*m), comparable in size to fine sand grains (100–500 *µ*m), disrupt uniform packing and increase pore-scale heterogeneity. Along with the observed increase in hydraulic conductivity (Fig. 3a), this results in greater variability in flow velocities, thereby increasing mechanical dispersion and causing a broader breakthrough curve (Fig. 4d). However, at higher concentration of 5%, PVC fills voids more uniformly which may reduce pore-scale heterogeneity and promote more uniform flow paths. This potential reduction in pore connectivity can decrease flow variability and mechanical dispersion, leading to a narrower BTC compared to 2%, though still more spread than in pure sand. We observed a pronounced shift of the BTC in Fig. 4e when 5% PVC was added to the medium sand. However, in coarse sand (Fig. 4f), the BTC remained similar to that of the pure sample, regardless of PVC concentration.

Our analysis across all sandy soils (fine, medium, and coarse) and microplastic types and concentrations (PE and PVC) indicates that the presence of MPs generally leads to an early breakthrough and a delay in reaching equilibrium (the plateau of the BTC). These findings primarily mark the alteration of pore spaces by MPs which enhance the flow through the newly formed (preferential) channels, and retard the transport of the solute with formation of larger stagnant regions. With the formation of micro-retention zones, solutes can temporarily diffuse into low-flow regions before rejoining the main advective stream. This mechanism extends the breakthrough curve and resembles solute transport behavior in dual-porosity or mobile-immobile systems, which are common in heterogeneous porous media^55^. The observed spread in the breakthrough curve at high concentrations of PE and PVC (particularly in Fig. 4b and e) can be attributed to the formation of these immobile zones. As the brine moves through the mobile domain, a portion of the solute diffuses into stagnant regions. Over time, the solute slowly diffuses back into the mobile zone and leads to a prolonged release of the tracer and thus an extended BTC.

**Fig. 5:**
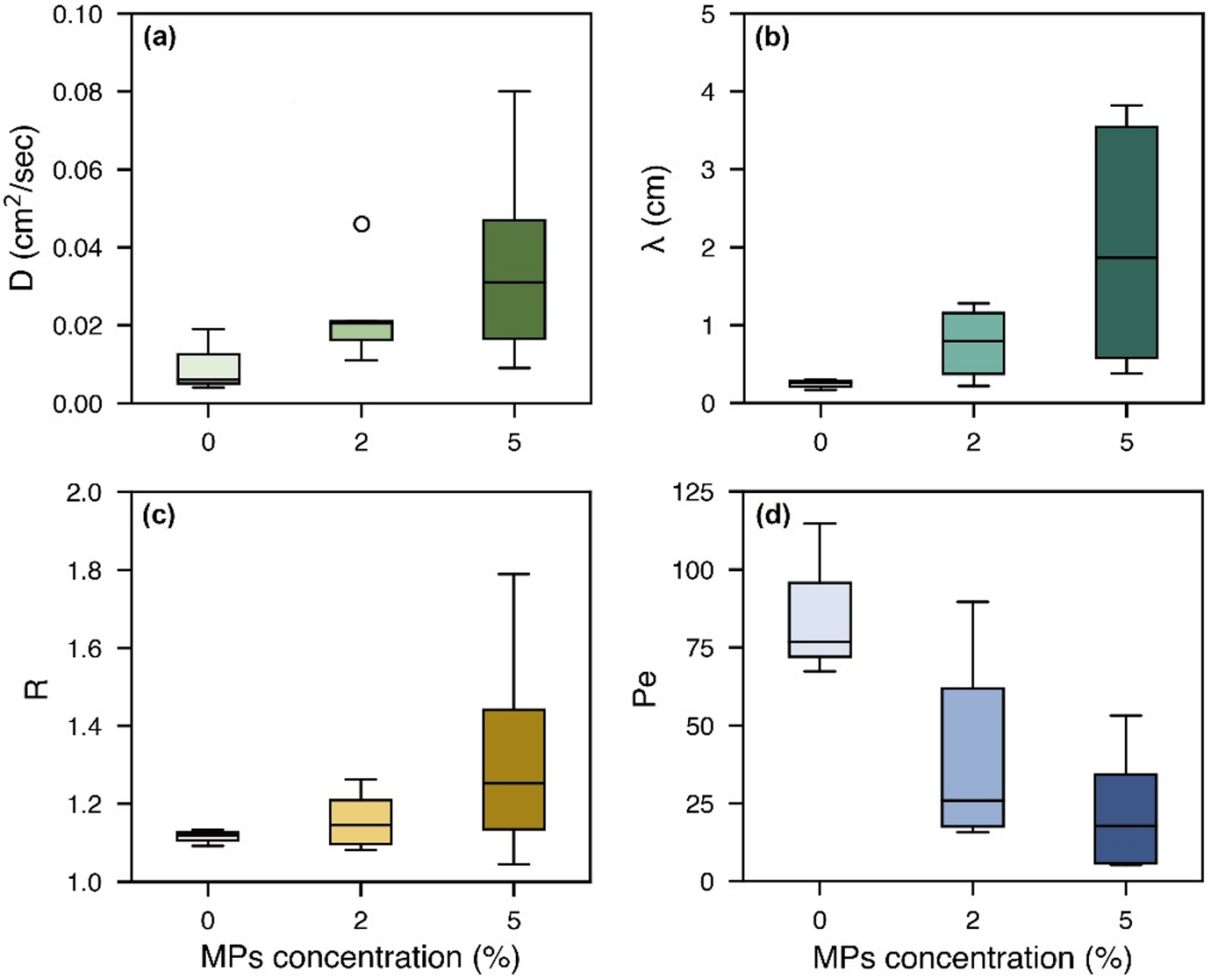
The impact of PE and PVC concentration on the variation of diffusion-dispersion coefficient (**a**), longitudinal dispersivity (**b**), retardation factor (**c**), and Peclet number (**d**) across all sandy soils (fine, medium, and coarse).

MPs with particles smaller than those of sandy soil can partially occupy pore spaces and, in some cases, clog pore throats, as illustrated in Fig. 2c^56^. Similar to the impact of electrostatic interactions on saturated hydraulic conductivity, smaller and more stable PE particles distribute more uniformly within the pore network, enhancing clogging of fine pores. An increase in the ionic strength of the pore solution (with the brine tracer) further affects the mobility of MPs by altering the zeta potential of both MPs and sandy soil. According to the Derjaguin-Landau-Verwey-Overbeek (DLVO) theory, increased salinity compresses the electrical double layer, which reduces the effective range of electrostatic repulsion. This compression leads to enhanced van der Waals attraction, promoting MP aggregation and deposition within pore spaces^57,58^. As MP concentration increases, these obstructions form additional micro-barriers that modify the pore structure and disrupt the continuity of flow paths. The restructured pore network promotes the development of preferential flow paths (where water moves rapidly) and stagnant or low-velocity zones that induce more divergent and tortuous flow paths^59^. This results in greater heterogeneity in pore-scale water velocities and, consequently, an increase in hydrodynamic dispersion and longitudinal dispersivity with increase in MP concentration (Fig. 5).

Our modeling results in Fig. 5c indicate that the retardation factor slightly increases with rising microplastic concentrations (see details in Table 3). Since NaCl is a conservative tracer, this increase is likely due to physical alterations in the porous medium (primarily the porosity). Specifically, increased flow path tortuosity delays solute transport and slows advective flow, as reflected in the lower Peclet numbers in Fig. 5d.

**Table 3:** Variation of retardation factor (R), diffusion-dispersion coefficient (D), pore velocity (*v*), Peclet number (Pe), and longitudinal dispersivity (λ) with various concentrations of PE and PVC, along with the corresponding ADE model performance metrics (R^2^ and RMSE).

	MPs	*R*	D (cm^2^/sec)	v (cm/sec)	Pe	λ (cm)	*R* ^2^	RMSE
Fine sand	Pure sand	1.13	0.004	0.015	76.83	0.26	0.997	0.005
	2% PE	1.26	0.015	0.012	15.66	1.28	0.989	0.010
	5% PE	1.31	0.018	0.005	5.37	3.72	0.979	0.003
	2% PVC	1.22	0.021	0.017	16.22	1.23	0.990	0.006
	5% PVC	1.19	0.009	0.016	36.11	0.55	0.995	0.006
Medium sand	Pure sand	1.09	0.006	0.035	114.76	0.17	0.999	0.005
	2% PE	1.09	0.021	0.032	30.28	0.66	0.997	0.003
	5% PE	1.48	0.048	0.013	5.23	3.82	0.990	0.006
	2% PVC	1.10	0.046	0.049	21.58	0.93	0.994	0.003
	5% PVC	1.79	0.016	0.042	53.24	0.38	0.992	0.006
Coarse sand	Pure sand	1.12	0.019	0.063	67.36	0.30	0.998	0.002
	2% PE	1.08	0.011	0.050	89.72	0.22	0.997	0.007
	5% PE	1.05	0.080	0.026	6.58	3.04	0.993	0.001
	2% PVC	1.19	0.020	0.071	72.41	0.28	0.998	0.003
	5% PVC	1.12	0.044	0.064	29.09	0.69	0.997	0.006

### Limitations and future developments

This study provides mechanistic insights into how microplastics alter pore structure, subsequently affecting hydraulic conductivity and solute transport in soil. While our findings enhance the understanding of microplastic-soil interactions under controlled conditions, further research is needed to elucidate their long-term effects in natural soils, particularly in the presence of organic matter and microbial communities.

We used microplastics smaller than or comparable in size to the sand grains; therefore, the observed reduction in hydraulic conductivity primarily reflects pore filling and clogging effects. While these findings are most applicable to situations where MPs are fine relative to soil particles, future work should examine cases where larger particles act as structural elements that modify pore spaces and potentially enhance hydraulic conductivity, as conceptually illustrated in Fig. 3b. Future studies could further incorporate inert particle controls such as fine quartz or glass beads to more clearly distinguish the intrinsic chemical effects of microplastics (e.g., hydrophobicity) from pore-scale physical mechanisms (e.g., pore clogging). Our solute transport experiments employed a conservative tracer (brine); however, the interactions between reactive solutes and microplastics require further investigation. Additionally, expanding the study to include various plastic types at different degradation stages will be essential for developing more comprehensive models of microplastic behavior under real environmental conditions.

One fruitful extension of this work would be to combine high-resolution three-dimensional imaging (confocal microscopy or X-ray) experiments and modeling to develop tools to predict structural and transport behavior of soils containing microplastics. Pore network models could be extracted from images to represent the topology and connectivity of different soils, coupled with transport codes to predict dispersion, interpret the long delay in reaching to equilibrium, and the dual porosity behavior observed here^60,61^. This would then allow the assessment of behavior outside the range studied directly by experiment.

## Conclusions

We investigated the influence of microplastics on water and solute transport dynamics in sandy soils. Fluorescence and confocal microscopy of microplastic distribution in synthesized porous microchips revealed that MPs primarily alter pore structure by clogging pore throats, thereby disrupting pore network connectivity. This structural alteration typically results in reduced hydraulic conductivity and impaired transport properties within the soil profile. Overall, our results showed reductions in saturated hydraulic conductivity of 35% and 67% in fine sand, 39% and 74% in medium sand, and 18% and 58% in coarse sand for samples containing 5% PVC and 5% PE, respectively. PE particles, which were considerably smaller than the sand grains, exerted a more pronounced effect on flow reduction. At a concentration of 5%, PE particles effectively filled pore spaces and impeded water movement through the soil. The comparable zeta potential of PE particles (− 35.43 mV) and sand grains (− 15.84 to − 26.45 mV) increases particle stability in the soil matrix, leading to enhanced clogging of fine pores.

Monitoring of solute transport in soil column demonstrated that changes in pore structure increase flow path tortuosity and heterogeneity that, consequently, broaden breakthrough curves. Our findings indicated early breakthrough of the solute, attributed to the development of preferential flow paths that facilitate transport of the tracer through the soil column. On the other hand, the observed delay in solute transport and spread of the breakthrough curve can be explained by the formation of immobile zones, where a portion of the solute diffuses into stagnant regions before gradually returning to the mobile zone. Higher MP concentrations and changes in solution chemistry (e.g., increased salinity) enhance aggregation and clogging of microplastics in pore spaces leading to greater mechanical dispersion, reduced flow connectivity, and higher retardation in solute transport process in porous media.

A comprehensive understanding of how microplastics affect soil functions and its ecological processes is essential to protect the integrity of terrestrial ecosystems. Our findings demonstrate that microplastic contamination can significantly modify soil hydraulic conductivity and solute transport behavior leading to altered water infiltration, preferential flow development, and changes in nutrient and agrochemical mobility. These effects have direct practical implications for irrigation efficiency, fertilizer delivery, and contaminant leaching in agricultural systems, especially in soils affected by plastic mulching, wastewater irrigation, or amendments such as sewage sludge and compost. The insights provided in this study can improve the predictive modeling of microplastic fate in soil and contribute in shaping informed strategies to mitigate the adverse impacts of microplastic contamination on soil health and agricultural productivity.

## Data Availability

All data supporting the findings of this study are available within the article.
